# Measurements of an Effective Longitudinal Coherence Length in Transmission Small Angle X-ray Scatterings

**DOI:** 10.3390/nano10081549

**Published:** 2020-08-07

**Authors:** Chun-Ting Liu, Bo-Ching He, Guo-Dung Chen, Alice Chinghsuan Chang, Wen-Li Wu, Wei-En Fu

**Affiliations:** Nano and Semiconductor Metrology Laboratory, Center for Measurement Standards, Industrial Technology Research Institute, Hsinchu 30011, Taiwan; itriA60470@itri.org.tw (C.-T.L.); hopc@itri.org.tw (B.-C.H.); Eric_chen@itri.org.tw (G.-D.C.); Alice.C.Chang@itri.org.tw (A.C.C.); wenli.nist@gmail.com (W.-L.W.)

**Keywords:** effective longitudinal coherence length, transmission small-angle X-ray scattering, integrated circuit, semiconductor devices, X-ray metrology

## Abstract

The notion of an effective longitudinal coherence length with its value much greater than $\lambda^{2}/\left(2\Delta \lambda \right)$ has been adopted in small-angle X-ray scattering communities for years, where λ and Δλ denote the incident wavelength and its spread, respectively. Often the implications of the effective longitudinal coherence length do not even enter considerations in the designing and data treatment of small-angle scattering experiments. In this work, conventional transmission small-angle X-ray scattering (tSAXS) was performed to reveal a clear angular dependence on effective longitudinal coherence length. The measured values of effective longitudinal coherence length can be as high as one millimeter, whereas the value of calculated $\lambda^{2}/\left(2\Delta \lambda \right)$ is in nanometers.

## 1. Introduction

In light of an ever-shrinking feature size and the increasing complexity of 3D architecture of today’s integrated circuit (IC) devices, the need for metrology tools with a sub-nanometer resolution and a great penetration power has been a major challenge for IC chip manufacturers. Currently, the feature size reaches 3 nm, and 3D architecture, such as gate-all-around (GAA) transistors, is becoming a reality to replace tri-gate field-effect transistors (FinFET) [1,2]. Among possible metrology solutions under development, X-ray-based methods have been identified, as early as 2007 in an International Technology Roadmap for Semiconductors (ITRS) roadmap [1], as potential solutions for measuring nanoscale features because of their sub-nanometer resolution and great penetration power.

Transmission small-angle X-ray scattering (tSAXS), one of the X-ray based methods, has been extensively studied to determine its 3D feature dimensions even in high-aspect-ratio nanostructures. Most work has been performed using a synchrotron X-ray source for its high-beam flux or high brilliance, which enables tSAXS measurements of samples with a minuscule scattering volume [3,4,5,6]. However, synchrotron sources are simply too large and too expensive for daily industrial deployment. A modern high-brilliance laboratory X-ray system equipped with a liquid-metal jet X-ray generator has been successfully demonstrated for tSAXS applications by the National Institute of Standards and Technology (NIST) [2], but the measurement speed is far too slow for inline metrology applications in IC fabrications.

The tSAXS method is identical to the conventional small-angle X-ray scattering (SAXS) technique widely used in materials science, biology and many other sectors. However, for future applications as a metrology tool in IC fabrications, SAXS in a reflection mode, i.e., rSAXS, has also been proposed and studied [7]. To avoid possible confusion, the letter “t” is added in front of SAXS throughout this work. To overcome the difficulty of weak tSAXS signals using conventional laboratory X-ray sources, a potential remedy has been proposed [8], wherein an enhancement object with a strong scattering cross-section is positioned adjacent to the target object within an effective longitudinal coherence length. The scattering intensity from the target is expected to increase by adding the interaction contribution between the target and the enhancement objects. In this work, the magnitude of effective longitudinal coherence length was measured over a range of scattering angles via a simple tSAXS experiment where a nanoscale line grating was chosen as the target object.

**Figure 1:**
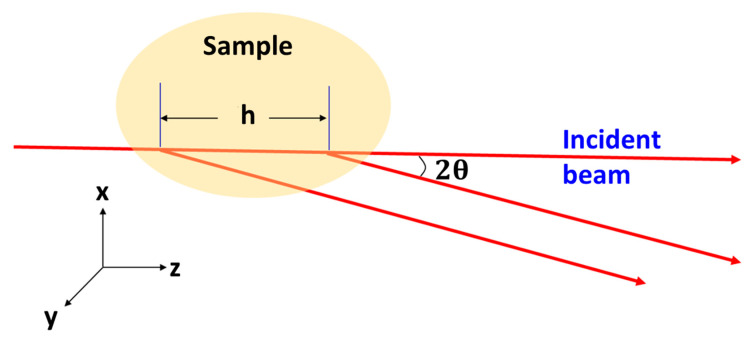
A schematic transmission small-angle X-ray scattering (tSAXS) diagram highlights the approximations invoked in typical small-angle scatterings. An incident beam with an infinitesimal cross-section and the scattered beams from two points within the sample are parallel as seen by the detector.

As explained in the work by Sinha et al. [9], the effective longitudinal coherence length is directly related to the difference in the path length as the X-ray traverses and is scattered by different parts of the sample. The path length is a sum of the distance from the source to the sample location where the scattering occurs and from this sample location to the detector. The above-mentioned work was aimed at addressing the combined resolution and coherence challenges in surface reflection X-ray measurements. It also pointed out an important difference in the manifestations between the instrument resolution and the coherence length. In this work, the effect of longitudinal coherence length on the scattering intensities is investigated while the angular resolution stays unchanged. A simplified tSAXS geometry is depicted in Figure 1. In this simplification, both the source aperture-to-sample and sample-to-detector distances are much larger than the aperture size or the incident beam cross-section. In addition, the scattering angle 2θ is small enough so that the scattering vector component along the incident direction, $q_{z}=\left(4\pi /\lambda \right)sin^{2}\theta$, can be ignored.

As illustrated in Figure 1, the scattered beams at angle 2θ from two points within the sample with a distance h along the incident beam direction have a difference in path length once they reach the detector, where the path length difference is simply h(1−cos(2θ)). This path length difference needs to be less than the longitudinal coherence length of the incident beam given as $\xi =\lambda^{2}/\left(2\Delta \lambda \right)$, where λ and Δλ denote the incident wavelength and the wavelength spread, respectively. Explicitly, the above statement can be expressed as:

(1) $$h\ll \xi /\left(1-\cos2\theta \right)\approx \xi /2\theta^{2}$$

The last term, $\xi /2\theta^{2}$, denotes the effective longitudinal coherence length ξ′. A similar definition has also been mentioned by Mochrie et al. in their work on X-ray reflection intensity fluctuation from block copolymer surfaces [10].

The present work is aimed at measuring the effective longitudinal coherence length and its dependence on the scattering angle via a set of tSAXS experiments. The results of this work will provide a guideline for selecting the appropriate distance between the target and the enhancement objects and, hence, lay the foundation for pursuing the idea of tSAXS signal enhancement of nanostructured samples encountered in semiconductor fabrications.

## 2. Methods

**Figure 2:**
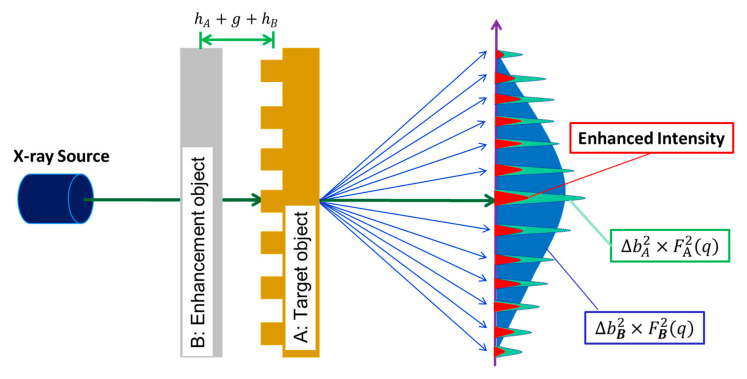
A schematic diagram of the tSAXS experiments for measuring the effective longitudinal coherence length. A and B denote the target grating and enhancement objects, respectively, with a gap g between them. The value of g is adjusted by inserting spacers with different thicknesses.

A silicon line grating was used as the target (A), and an amorphous carbon disc (B) with a strong scattering cross-section was used as the enhancement object, as shown in Figure 2. The gap g between these two objects was adjusted between micrometers and tens of millimeters by spacers with different thicknesses. The scattering intensities from the target grating can be described as [11,12]:

(2) $$I_{A}\propto \Delta b_{A}^{2}\times F_{A}^{2}\left(q\right)$$

where $\Delta b_{A}$ is a contrast factor of the target grating and proportional to the electron density of silicon, $F_{A}\left(q\right)$ is the Fourier transform of the target grating, and q is the scattering vector defined in its typical way. Due to the symmetry of the grating used, only the real component exists in its Fourier transform.

National Institute of Standards and Technology Standard Reference Materials (NIST SRM) 3600 [13] (Material Measurement Laboratory, National Institute of Standards and Technology, Gaithersburg, MD, USA), which is an amorphous carbon disc, was chosen as the enhancement object B. It was chosen due to its ample scattering intensities covering an angular region similar to the target grating. After the addition of the amorphous carbon disc to the target grating, the scattering intensities ($I_{AB}$) of the composite sample, under the assumption of a complete coherence between these two objects, became:

(3) $$\begin{array}{cc} I_{AB}\left(q\right) & ={\left[\Delta b_{A}\times F_{A}\left(q\right)+\Delta b_{B}\times F_{B}\left(q\right)\right]}^{2}\\ & =\Delta b_{A}^{2}F_{A}^{2}\left(q\right)+\Delta b_{B}^{2}F_{B}^{2}\left(q\right)+2\Delta b_{A}\Delta b_{B}F_{A}\left(q\right)F_{B}\left(q\right) \end{array}$$

where $2\Delta b_{A}\Delta b_{B}F_{A}\left(q\right)F_{B}\left(q\right)$, denoted as the interaction term between the target grating (A) and the enhancement object (B), is the origin of signal enhancement over $\Delta b_{A}^{2}\times F_{A}^{2}\left(q\right)$. The effect of a finite effective longitudinal coherence length ξ′ is expected to decrease this interaction contribution by a factor described as:

(4) $$exp\left(-\left(h_{A}+g+h_{B}\right)/\xi^{\prime }\right)$$

The quantity $D=h_{A}+g+h_{B}$ is the mean distance between the target grating A and the enhancement object B, as shown in Figure 2. Given that the height, $2h_{A}$, of the grating is 110 nm, the thickness, $2h_{B}$, of NIST SRM 3600 is 1.055 mm, and a polyimide film of 10 μm thickness is placed on the grating surface for protection, this configuration led to a minimum distance of D at 537 µm. With hollow metal spacers, this distance was increased incrementally up to 12,120 µm in the tSAXS experiments.

The exponential form of Equation (4) has been used by others, such as Equation (17) of Sinha et al. [9] and references therein, to account for the effect of ξ on the interference between two points along the incident beam path. However, in the present work, the mean distance between the target grating and the enhancement object is placed as the nominator instead of the distance between two points along the incident beam path. Given the thickness of these two objects at 110 nm and 1.055 mm, Equation (4) implies that the interaction between the scattering from these two objects can be approximated by their center positions. This is a highly simplified approximation, and hence, Equation (4) can be considered as an empirical one to guide the experiments and the data treatment.

It is also noteworthy that both Equations (2) and (3) imply that the first Born approximation, or the kinematic scattering model, is invoked in this work; i.e., no multiple scattering events are considered. In addition, a typical small-angle scattering approximation of negligibly small $q_{z}$ is also invoked, while the *z*-axis denotes the incident beam direction. Due to this approximation, the interaction term appearing in Equation (3) does not contain exp (−$iDq_{z}$) explicitly, where the quantity D is defined previously as the mean distance between the target and the enhancement objects.

For the synchrotron source used in this work, the X-ray longitudinal coherence length, ξ, was estimated conventionally to be about ~4.13 nm using $\xi =\lambda^{2}/\left(2\Delta \lambda \right)$, where λ is the X-ray wavelength at 0.0832 nm and Δλ is the X-ray wavelength spread of 0.000832 nm [14]. Given that the scattering angle 2θ is far less than unity in tSAXS, the magnitude of ξ′, defined as $\xi /2\theta^{2}$, is expected to be larger than 4.13 nm by over several orders of magnitude. By positioning an enhancement object within the effective longitudinal coherence length from the target grating, the scattering intensity of the composite sample is expected to increase. The extent of increase depends on the distance D as well as the scattering angle 2θ.

## 3. Experimental Results and Discussions

The tSAXS experiments for the evaluation of the effective longitudinal coherence length were performed at beamline TLS 23A of the National Synchrotron Radiation Research Center (NSRRC) of Taiwan. The incident X-ray beam after a double multilayer Mo-based B4C monochromator has the following parameters: the photon energy is 15 keV (or the wavelength is 0.083 nm), the energy resolution (ΔE/E) is ~0.01, the flux is ~10^10^ photons/sec, and the beam size = 0.5 mm × 0.6 mm. The distance from the composite sample to the detector is 4,632 mm. The target grating, which is the silicon line grating with 278 nm pitch etched from a silicon single crystal substrate and 110 nm in grating height, was used as the target grating (A), as shown in Figure 2. The enhancement object thickness of NIST SRM 3600 (B) is 1.055 mm, and a polyimide film of 10 µm thickness is placed on the grating surface. The scattering intensity of the composite sample for the experiments was expected to be less than that of the theoretical calculation due to the absorption of scattering intensity by the substrate of the target grating and enhancement object. Therefore, it was necessary to measure the transmission coefficient of both the target grating and the enhancement object for correcting the observed scattering intensity of the composite samples. The X-ray transmission coefficient $T_{A}$ for the target grating was measured with a result of 17.73% and 88.27% for $T_{B}$ for NIST SRM 3600. The transmission coefficient of the composite sample shown in Figure 2 is, then, calculated as the product of $T_{A}$ and $T_{B}$. The resultant scattering intensities $I_{A}$ from the target grating were multiplied by $T_{B}$, and the scattering intensities $I_{B}$ from the NIST SRM were multiplied by $T_{A}$ before they were presented and analyzed with $I_{AB}$ from the composite sample.

**Figure 3:**
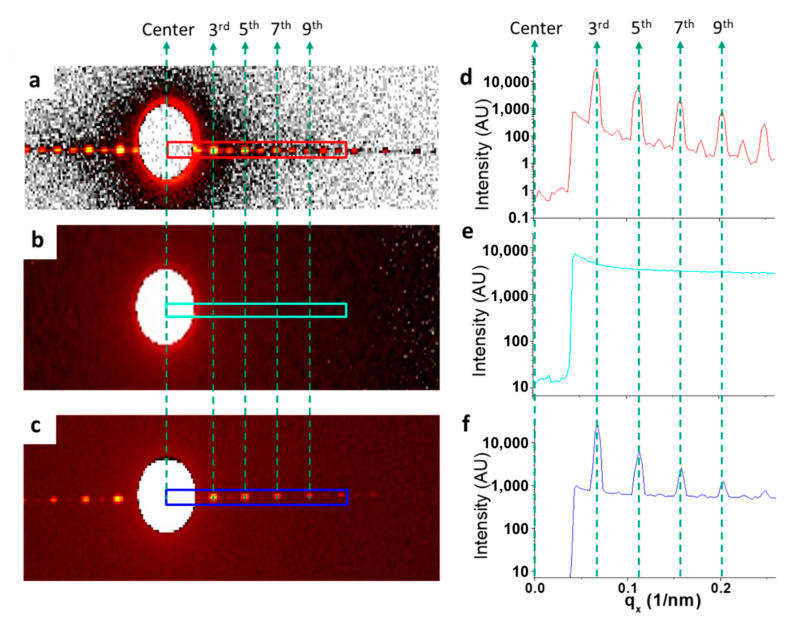
The scattering patterns recorded on a 2D detector of Si grating, National Institute of Standards and Technology Standard Reference Materials (NIST SRM) 3600, and the composite sample at a separation distance of 537 µm are given in (**a**–**c**), respectively. The corresponding averaged intensities are given in (**d**–**f**), respectively. For (**d**–**f**), the intensities presented were from their sector average.

The scattering intensity of the composite sample was measured at the first four observed intense peak positions, as shown in Figure 3. Since the line width and repeat of target grating (line pitch) are close to 1:2, this results in weak scattering intensities on all peaks with even orders. With the first scattering order behind the beam stop, only the intensities from the third, fifth, seventh and ninth peaks were used for analysis [11]. The signals after the ninth order were rather weak and hence are not included in the discussions.

**Figure 4:**
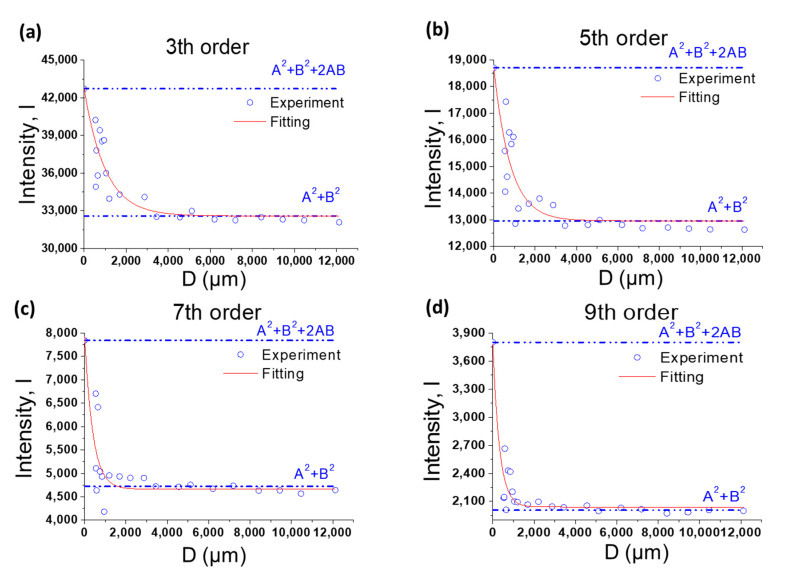
The fitting of the experiment scattering intensities of 3rd (**a**), 5th (**b**), 7th (**c**), and 9th (**d**) peaks. The intensity of D = 0 is calculated by the value of $\left(A^{2}+B^{2}+2AB\right)$. The red dotted line represents an exponential fit of the data following Equation (3) to estimate the effective longitudinal coherence length $\xi^{\prime }$.

The distance D between the Si target grating and NIST SRM 3600 is the sum of the half grating height ($h_{A}$), the gap (g) between Si line grating and the NIST SRM 3600 and the half thickness of NIST SRM 3600 ($h_{B}$). Different D values were altered by using different thicknesses of hollow metal spacers of the different g values. The scattering intensities $I_{AB}\left(\theta_{x}\right)$ of the composite samples measured at the different values of D are given in Figure 4 for all four peak positions. With a finite effective longitudinal coherence length $\xi^{\prime }\left(\theta \right)$, Equation (3) can be rewritten as:

(5) $$I_{AB}=\left(I_{A}{}^{2}+I_{B}{}^{2}\right)+\left(2I_{A}I_{B}\right)\times exp\left(-\frac{D}{\xi^{\prime }\left(\theta \right)}\right)$$

The variables $I_{A}{}^{2}$ and $I_{B}{}^{2}$ stand for the scattering intensities from the target grating and NIST SRM 3600 after being normalized with their transmission coefficients. Given that both $F_{A}\left(q\right)$ and $F_{B}\left(q\right)$ are real due to the symmetry of the sample geometry, the square root of the observed intensities from the silicon line grating and NIST SRM 3600 were taken as $I_{A}$ and $I_{B}$, respectively. For simplicity, $I_{A}$ and $I_{B}$ are replaced by symbols A and B in the rest of this work. In Figure 4, the values of both $\left(A^{2}+B^{2}+2AB\right)$ and $\left(A^{2}+B^{2}\right)$ were given in addition to the observed scattering intensities from the composite sample at four different peak positions as a function of D. Based on Equation (4), $\left(A^{2}+B^{2}+2AB\right)$ and $\left(A^{2}+B^{2}\right)$ are the asymptotic values of the scattering intensities of the composite sample as D approaches zero and infinity, respectively. The best-fit values $\xi^{\prime }\left(\theta \right)$ at scattering angle 2θ corresponding to the third, fifth, seventh and ninth scattering peaks are summarized in Table 1 together with the calculated value of $\xi^{\prime \prime }$ using the relation $\xi^{\prime \prime }=2\theta^{2}\xi^{\prime }\left(\theta \right)$.

The best-fit effective longitudinal coherence length, $\xi^{\prime }\left(\theta \right)$, at all four peak positions is significantly greater than 4.13 nm, calculated from $\xi =\lambda^{2}/\left(2\Delta \lambda \right),$ by several orders of magnitude. In addition, the values for $\xi^{\prime }\left(\theta \right)$ exhibit an angular dependence that seems to follow the trend given in $\xi^{\prime }\left(\theta \right)=\xi /2\theta^{2}$. The resulting $\xi^{\prime \prime }$ values calculated as $\xi^{\prime \prime }=2\theta^{2}\xi^{\prime }\left(\theta \right)$ are also given in the above table. All the calculated values are less than the calculated ξ of 4.13 nm, and the discrepancy seems to decrease as the peak order increases. This indicates that the small-angle scattering approximation of h(1−cos(2θ)) for the beam path length difference by itself is insufficient to account for the observed loss in the longitudinal coherence, so other factors must play a role. The magnitude and the angular dependence of the discrepancy between the observed $\xi^{\prime }\left(\theta \right)$ and what is predicted as $\xi^{\prime }\left(\theta \right)=\xi /2\theta^{2}$ seems to suggest that a finite angular source size seen by samples will provide the correct answer, at least qualitatively. This is the very factor dictating the transverse coherence length; however, as pointed out explicitly by Petukhov et al., this finite source angle also plays an important role in the loss of longitudinal coherence [15]. In other words, both the wavelength spread and the angular source size of the light source seen by the sample will contribute to the path difference of the beams scattered at various points within the sample. The effect of this finite source size has also been discussed by Sinha et al. [9] as the finite aperture effect on the beam path difference. Our result of $\xi^{\prime \prime }$ given in Table 1 indicates that, up to the ninth peak position, the contribution of a finite angular source size to the loss of longitudinal coherence prevails over the contribution from the wavelength spread. Without precise knowledge of the angular source size of the experimental set-up at this moment, a quantitative calculation of the value of $\xi^{\prime }\left(\theta \right)$ including both the wavelength spread and the finite source size will be performed in the future.

It is noteworthy that the thickness of NIST SRM 3600 is 1.055 mm and the q range covers 0.008 Å^−1^ to 0.25 Å^−1^ for an X-ray wavelength of 1.0332 Å [13]. This q range, therefore, corresponds to a range of 2θ from 1.32 × 10^−3^ to 4.12 × 10^−2^ in rad. This range is far greater than the ninth-order peak with a 2θ position of 0.0027 reported in this work, where the value of $\xi^{\prime }$ is 345.9 μm, a value far less than the sample thickness of 1.055 mm. It is useful to look into the possible effect of longitudinal coherence length on the observed SRM scattering intensities, especially in the high angle region where the value of $\xi^{\prime }$ continues to decrease. Along the beam path of 1.055 mm through the sample thickness, the scatterings from different parts may not interact coherently, and the extent of coherent interaction depends on the distance between these parts. For simplicity, one can consider the sample thickness of 1.055 mm as D in Figure 4, where the data indicate that only a partial coherence exists at D= 1.055 mm for all the peak positions investigated. It is also noteworthy that the wavelength spread was ~1 × 10^−3^ for the ultra-small-angle X-ray scattering (USAXS) instrument used by Allen et al. to calibrate SRM 3600 instead of ~1 × 10^−2^ for the present work. A narrow wavelength spread will result in an increase in ξ; however, in a high angular range of 2θ, the value of $\xi^{\prime }$ can become comparable or even less than what was encountered in this work. The above discussion suggests that the SAXS measurement results by different laboratories for SRM 3600 can vary depending on the incident beam wavelength and its spread, aperture size, source-to-sample and sample-to-detector distances. In addition, the extent of variations from what was reported [13] can also depend on the angular range of the measurement results.

## 4. Conclusions

A scheme for measuring the effective longitudinal coherence length, $\xi^{\prime }$, encountered in conventional transmission small-angle X-ray scattering (tSAXS) measurements was devised. The experiments yield values for $\xi^{\prime }$ far greater than the calculated one based on $\xi =\lambda^{2}/\left(2\Delta \lambda \right)$, where λ and Δλ stand for the wavelength and wavelength spread, respectively. The measured values of $\xi^{\prime }$ can be as high as one millimeter, whereas the value of $\lambda^{2}/\left(2\Delta \lambda \right)$ is in nanometers. The measured $\xi^{\prime }$ exhibits a strong angular dependence, and it decreases with scattering angle 2θ; however, the extent of decrease is less than what is dictated by $\xi^{\prime }\left(\theta \right)=\xi /2\theta^{2}$, especially at low angles. The above relation between $\xi^{\prime }$ and ξ was rationalized through simple geometrical reasoning by considering only the wavelength spread. The contribution to the beam path length difference due to a finite aperture size or a finite angular source size was not included in deriving the above relation. One expects that the fit between the experimental values of $\xi^{\prime }$ and the theoretical one will improve once the finite angular source size effect is included. The implications of the observed magnitude and the angular dependence of $\xi^{\prime }$ are discussed for tSAXS measurements from samples with their thickness in millimeters, a thickness range not uncommon for polymeric and organic materials.

## 5. Patents

The US patent US10352694B2: Contactless dual-plane positioning method and device resulting from the work reported in this manuscript.

## Figures and Tables

**Table 1:** The scattering intensities $A^{2}$, $B^{2}$ of the grating sample and the NIST SRM 3600, respectively, the calculated values for 2AB, the peak position in scattering angle, the best-fit values of the effective longitudinal coherence length, and resulting values for $\xi^{\prime \prime }$ are listed for four peak positions.

Scattering Order	*A*^2^ + *B*^2^	2*AB*	2*θ* (Rad)	$\xi^{\prime }\;(\mu m)$	$\xi^{\prime \prime }\;(\mathrm{nm})$	R^2^
3rd	32,591	10,127	0.0009	1,002	0.406	0.83
5th	12,965	5,735	0.0015	799.6	0.900	0.67
7th	4,723	3,120	0.0021	383.5	0.846	0.67
9th	2,006	1,795	0.0027	345.9	1.261	0.87
